# Supplementary material for: Intended and experienced literacy practices in a Swedish undergraduate nursing education
Source: PLoS One. 2025 Oct 24;20(10):e0335166. doi: 10.1371/journal.pone.0335166 (PMC12551825; doi:10.1371/journal.pone.0335166)
Supplement: S1 File — (PDF) [file pone.0335166.s001.pdf]

Form 1

Record ID

____

Bakgrundsfrågor

Datum

____

Vilken är din ålder (skriv ålder i heltal med siffror, t ex 24)

____

Vilken är din högst genomförda utbildning?

◯ Grundskola
◯ Gymnasium
◯ Universitet/högskola
◯ Annat

Vilken form har din utbildning i huvudsak?

◯ Campusförlagd utbildning
◯ Distansutbildning

Vilken grupptillhörighet har du?

◯ A
◯ B

Hur och varför studenter antecknar vid och kring föreläsningar

Skriver du egna anteckningar under föreläsningar?

◯ Ja, alltid
◯ Ja, ibland
◯ Ja, sällan
◯ Nej

Vad är ditt syfte med att anteckna?

Exempel på svar från studenter på andra utbildningar har tex varit att man vill minnas, att man lär sig av att anteckna, att man vill förtydliga svåra ord eller begrepp, etc.

Vad är ditt syfte med att anteckna?

Exempel på svar från studenter på andra utbildningar har tex varit att man vill minnas, att man lär sig av att anteckna, att man vill förtydliga svåra ord eller begrepp, etc.

Varför antecknar du ibland och inte alltid?

Exempel på svar från studenter på andra utbildningar har t ex varit att handoutsen är så bra att det inte behövs, vissa föreläsare säger inget mer än det som redan står på handoutsen, vissa pratar så fort att jag inte hinner, etc.

Vad är ditt syfte med att anteckna?

Exempel på svar från studenter på andra utbildningar har tex varit att man vill minnas, att man lär sig av att anteckna, att man vill förtydliga svåra ord eller begrepp, etc.

Varför antecknar du sällan och inte alltid?

Exempel på svar från studenter på andra utbildningar har t ex varit att handoutsen är så bra att det inte behövs, vissa föreläsare säger inget mer än det som redan står på handoutsen, vissa pratar så fort att jag inte hinner, etc.

Varför antecknar du inte?

Exempel på svar från studenter på andra utbildningar har t ex varit att handoutsen är så bra att det inte behövs, vissa föreläsare säger inget mer än det som redan står på handoutsen, vissa pratar så fort att jag inte hinner, etc.

Vilket eller vilka redskap använder du för att anteckna under föreläsningarna

	Alltid	Ibland	Sällan	Aldrig
Papper och penna	◯	◯	◯	◯
Dator eller surfplatta	◯	◯	◯	◯
Mobiltelefon	◯	◯	◯	◯
Kombination	◯	◯	◯	◯

Om du använder en kombination av redskap, vilken är din vanligaste kombination?
Om du inte svarat att du använder kombination så kan du hoppa över denna fråga.

Skriver du egna anteckningar efter föreläsningar?

◯ Ja, alltid
◯ Ja, ibland
◯ Ja, sällan
◯ Nej

Hur gör du detta?

◯ Individuellt ◯ Tillsammans med din grupp ◯ Tillsammans med vänner

Vilket eller vilka redskap använder du för att anteckna efter föreläsningarna?

	Alltid	Ibland	Sällan	Aldrig
Papper och penna	◯	◯	◯	◯
Dator eller surfplatta	◯	◯	◯	◯
Mobiltelefon	◯	◯	◯	◯
Kombination	◯	◯	◯	◯

Om du använder en kombination av redskap, vilken är din vanligaste kombination?
Om du inte svarat att du använder kombination så kan du hoppa över denna fråga.

Hur och varför gör studenter sammanställningar inför examinationer

Skriver du sammanställningar av dina anteckningar, kurslitteratur, handouts samt annat kursmaterial?

◯ Ja, alltid
◯ Ja, ibland
◯ Ja, sällan
◯ Nej

Vad är ditt syfte med att göra sammanställningar av dina anteckningar, kurslitteratur, handouts samt annat kursmaterial?

Exempel på svar från tidigare studenter har t ex varit att det är lättare att plugga inför en examination om allt är samlat, jag lär mig av att sammanställa, det blir tydligare om man kan lägga till bilder till anteckningar, etc.

Vilket eller vilka redskap använder du för att göra sammanställningar av dina anteckningar, kurslitteratur, handouts samt annat kursmaterial?

	Alltid	Ibland	Sällan	Aldrig
Papper och penna	◯	◯	◯	◯
Dator eller surfplatta	◯	◯	◯	◯
Mobiltelefon	◯	◯	◯	◯
Kombination	◯	◯	◯	◯

Om du använder en kombination av redskap, vilken är din vanligaste kombination?
Om du inte svarat att du använder kombination så kan du hoppa över denna fråga.

Hur gör du dina sammanställningar?

◯ Individuellt ◯ Tillsammans med din grupp ◯ Tillsammans med vänner

Varför gör du inga sammanställningar av dina anteckningar, kurslitteratur, handouts samt annat kursmaterial?

Exempel på svar från studenter på andra utbildningar har t ex varit att det inte behövs för att det tar onödig tid, jag lär mig bättre av att läsa än skriva, har ingen att samarbeta med, etc.

Undersökning om hur studenter stöttar varandra med anteckningar och sammanställningar

Hjälper du andra studenter med att anteckna?

	Alltid	Ibland	Sällan	Aldrig
Jag gör det på uppdrag för att någon behöver det	◯	◯	◯	◯
Jag gör det frivilligt till en vän/kurskamrat	◯	◯	◯	◯

Hjälper du andra studenter med att göra sammanställningar av anteckningar, kurslitteratur, handouts samt övrigt kursmaterial?

	Alltid	Ibland	Sällan	Aldrig
Jag gör det på uppdrag för att någon behöver det	◯	◯	◯	◯
Jag gör det frivilligt till en vän/kurskamrat	◯	◯	◯	◯

Fråga om du kan tänka dig att ställa upp på en intervju om dina anteckningar och/eller sammanställningar.

Om JA, erhålles 2 biobiljetter som tack för ditt deltagande.

Kan du tänka dig att bli intervjuad om dina anteckningar och sammanställningar

◯ Yes
◯ No

Skriv in din e-mailadress eller telefonnummer så att vi kan kontakta dig
