# Supplementary material for: Intended and experienced literacy practices in a Swedish undergraduate nursing education
Source: PLoS One. 2025 Oct 24;20(10):e0335166. doi: 10.1371/journal.pone.0335166 (PMC12551825; doi:10.1371/journal.pone.0335166)
Supplement: S2 File — (PDF) [file pone.0335166.s002.pdf]

Form 1

Record ID

____

Background questions

Date

____

What is your age (Enter your age as a whole number, e.g., 24)

____

What is your highest completed level of education?

◯ Elementary school
◯ High school
◯ University/college
◯ Other

What is the primary format of your education?

◯ Campus-based education
◯ Distance education

Which group do you belong to?

◯ A
◯ B

How and why students take notes during and around lectures

Do you take your own notes during lectures?

◯ Yes, always
◯ Yes, sometimes
◯ Yes, rarely
◯ No

What is your purpose for taking notes?

Examples from students in other programs include wanting to remember information, learning through note-taking, clarifying difficult terms or concepts, etc.

What is your purpose for taking notes?

Examples from students in other programs include wanting to remember information, learning through note-taking, clarifying difficult terms or concepts, etc.

Why do you sometimes take notes and not always?

Examples from students in other programs include that the provided handouts are sufficient, some lecturers only repeat what's on the handouts, lecturers speak too quickly, etc.

What is your purpose for taking notes?

Examples from students in other programs include wanting to remember information, learning through note-taking, clarifying difficult terms or concepts, etc.

Why do you rarely take notes and not always?

Examples from students in other programs include that the provided handouts are sufficient, some lecturers only repeat what's on the handouts, lecturers speak too quickly, etc.

Why don't you take notes?

Examples from students in other programs include that the provided handouts are sufficient, some lecturers only repeat what's on the handouts, lecturers speak too quickly, etc.

Which tools do you use for note-taking during lectures?

	Always	Sometimes	Rarely	Never
Paper and pen	○	○	○	○
Computer or tablet	○	○	○	○
Mobile phone	○	○	○	○
Combination	○	○	○	○

If you use a combination of tools, what is your most common combination?
(If you do not use a combination, you may skip this question)

Do you take your own notes after lectures?

○ Yes, always
○ Yes, sometimes
○ Yes, rarely
○ No

How do you do this?

◯ Individually ◯ With your group ◯ With your friends

Which tools do you use for note-taking after lectures?

	Always	Sometimes	Rarely	Never
Paper and pen	◯	◯	◯	◯
Computer or tablet	◯	◯	◯	◯
Mobile phone	◯	◯	◯	◯
Combination	◯	◯	◯	◯

If you use a combination of tools, what is your most common combination?
(If you do not use a combination, you may skip this question)

How and why students summarize course material before exams

Do you create summaries of your notes, course literature, handouts, and other course materials?

◯ Yes, always
◯ Yes, sometimes
◯ Yes, rarely
◯ No

What is your purpose for creating summaries of your notes, course literature, handouts, and other course materials?

Examples from previous students include that it is easier to study for exams if all material is organized, learning through summarizing, clarifying material by adding images, etc.

Which tools do you use to create summaries of your notes, course literature, handouts, and other course materials?

	Always	Sometimes	Rarely	Never
Paper and pen	◯	◯	◯	◯
Computer or tablet	◯	◯	◯	◯
Mobile phone	◯	◯	◯	◯
Combination	◯	◯	◯	◯

If you use a combination of tools, what is your most common combination?
(If you do not use a combination, you may skip this question)

How do you create your summaries?

◯ Individually ◯ With your group ◯ With your friends

Why don't you create summaries of your notes, course literature, handouts, and other course materials?

Examples from students in other programs include that it is unnecessary and time-consuming, better learning by reading than writing, lack of study partners, etc.

Survey about how students support each other with notes and summaries

Do you help other students by taking notes?

	Always	Sometimes	Rarely	Never
I do it upon request because someone needs it	◯	◯	◯	◯
I do it voluntarily for a friend/ classmate	◯	◯	◯	◯

Hjälper du andra studenter med att göra sammanställningar av anteckningar, kurslitteratur, handouts samt övrigt kursmaterial?

	Always	Sometimes	Rarely	Never
I do it upon request because someone needs it	◯	◯	◯	◯
I do it voluntarily for a friend/ classmate	◯	◯	◯	◯

Question about your willingness to participate in an interview about your notes and/or summaries.

If YES, you will receive 2 movie tickets as a thank you for your participation.

Would you be willing to be interviewed about your notes and summaries?

◯ Yes
◯ No

Please enter your email address or phone number so we can contact you.
