# Supplementary material for: Intended and experienced literacy practices in a Swedish undergraduate nursing education
Source: PLoS One. 2025 Oct 24;20(10):e0335166. doi: 10.1371/journal.pone.0335166 (PMC12551825; doi:10.1371/journal.pone.0335166)
Supplement: Attachment — Submitted filename: Review ReportPKreviewed290725.pdf [file pone.0335166.s004.pdf]

Review Report

for the Manuscript

on

Intended and experienced literature practices in a Swedish undergraduate nursing education

By

Dr Philip Kavan

1. The Abstract

The abstract appropriately summarized the manuscript. This includes objectives of the study, methods of data collection, analysis of the data, the results, and the findings. There are no discrepancies between the abstract and the remainder of the manuscript. By reading the abstract you already know what the content of the report is all about.

2. Introduction and review of literature

The introduction is concise, and the purpose of the study is clearly defined. The authors provided a rationale for performing the study by identifying a gap to fill in the knowledge gap based on a review of the literature and clearly stated the objective of the study. The articles in the literature reviewed were relevant.

3. **Materials and Methods Section**

The authors explained in detail the methods used to collect the data. Similarly, the description of the method used for analysis is clearly explained in detail. The methods are clear and easy for another researcher to reproduce the study using the same methods.

5. **The Results Section**

The findings were presented in tables and clearly explained in detail.

6. **The Discussion Section**

The discussion was concise. It captured the key findings and discussed them in detail in a systematic manner. Moreover, discussion was linked to literature. It also highlighted the strengths and limitations of the study.

7. **References**

An appropriate number of references were included in this paper. It was consistent with the style and properly formatted.

8**. General Comments**

Generally, the quality of this paper is academically sound. The quality of writing is acceptable. The structure of the paper was clearly stated with bold headings in the body of the paper.

I recommend this paper be accepted for publication.
