# Supplementary material for: Intended and experienced literacy practices in a Swedish undergraduate nursing education
Source: PLoS One. 2025 Oct 24;20(10):e0335166. doi: 10.1371/journal.pone.0335166 (PMC12551825; doi:10.1371/journal.pone.0335166)
Supplement: Attachment — Submitted filename: PONE-D-25-32643_reviewer _11082025.docx [file pone.0335166.s005.docx]

**Title:** Intended and experienced literacy practices in a Swedish undergraduate nursing education

Your manuscript explores the integration of academic and professional literacy practices in a Swedish undergraduate nursing program, combining curriculum analysis with a student survey on note-taking. The objectives of the study are clearly articulated, address a relevant gap, and employ an appropriate methodology. The language utilised is straightforward, though with minor errors, making the study accessible and comprehensible.

Nevertheless, the results fail to specify the extent, in numerical terms, to which participants demonstrate their ability to apply academic literacy, which is essential for evaluating the study's success. This data would undoubtedly provide the reader with a clearer understanding of the success rate of the literacy test conducted. To better grasp the findings, it would be beneficial to have precise definitions of academic and professional literacy.

Potential bias in student responses is noted: The response rate for the digital questionnaire was 40%, which may introduce bias if the characteristics or literacy practices of the responding students differ from those of non-responding students.

**To further enhance the study, consider:**

- Consider clarifying the sample and analysis methods for flow
- Assessing students' literacy skills: Incorporate objective evaluations of students' literacy skills to offer a more thorough insight into their capabilities.
- Exploring the impact on patient care: Examine how the incorporation of academic and professional literacy practices in nursing education influences patient care and outcomes.
- Longitudinal design: Contemplate a longitudinal approach to investigate how students' literacy practices develop throughout their nursing education and into their professional lives.

**Key Recommendations:**

- The language can be tightened for clarity and conciseness; some sentences are long and complex
- Break up long sentences and clarify definitions early in the text.
- Standardise terminology and proofread for typos and formatting issues.
- Reformat tables and ensure all data is clearly presented.
- Streamline the discussion to focus on the most important findings and their implications for nursing education.
- Make recommendations for curriculum development more direct and actionable in the conclusion

**Overall Comment:**

The manuscript is a valuable contribution to the field of nursing education. Furthermore, it presents original and relevant research, conducted and reported to a high standard. With minor revisions for language, formatting, and a stronger focus on practical implications, it will make a valuable contribution to the literature on literacy practices in nursing education and is suitable for publication.

**Some potential areas for future research include:**

- Evidence-Based Practice education: Analysing the effectiveness of evidence-based practice education programs in improving nursing students' literacy skills and competencies.
- Customisable Education Programs: Creating tailored educational programs that address diverse learning needs and encourage the integration of academic and professional literacy practices.
- Interprofessional Collaboration: Exploring the role of literacy practices in promoting interprofessional collaboration.
