# Supplementary material for: Intended and experienced literacy practices in a Swedish undergraduate nursing education
Source: PLoS One. 2025 Oct 24;20(10):e0335166. doi: 10.1371/journal.pone.0335166 (PMC12551825; doi:10.1371/journal.pone.0335166)
Supplement: Attachment — Submitted filename: Key Recommendations.docx [file pone.0335166.s006.docx]

**Key Recommendations:**

- The language can be tightened for clarity and conciseness; some sentences are long and complex
- Break up long sentences and clarify definitions early in the text.
- Standardise terminology and proofread for typos and formatting issues.
- Reformat tables and ensure all data is clearly presented.
- Streamline the discussion to focus on the most important findings and their implications for nursing education.
- Make recommendations for curriculum development more direct and actionable in the conclusion
